# Effects of 90- and 30-min naps or a 120-min nap on alertness and performance: reanalysis of an existing pilot study

**DOI:** 10.1038/s41598-023-37061-9

**Published:** 2023-06-18

**Authors:** Sanae Oriyama

**Affiliations:** Division of Nursing Science Graduate School of Biomedical and Health Sciences, Hiroshima University, Hiroshima, Japan

**Keywords:** Health care, Health occupations, Risk factors, Signs and symptoms

## Abstract

The aim of this study was to investigate alertness and cognitive performance immediately after and until the end of the night shift after taking a 120-min monophasic nap (One-nap) or a split 90-min and 30-min nap (Two-nap) during a 16-h simulated night shift, and the relationship between sleep quality and both alertness and performance. This study was performed in 41 females. Among them, 15 participants were included in the No-nap group, 14 in the One-nap group (22:00–00:00), and 12 in the Two-nap group (22:30–00:00 and 02:30–03:00). Participants were tested every hour from 16:00 to 09:00 for performance on the Uchida-Kraepelin test, as well as for subjective feelings of fatigue and drowsiness, body temperature, and heart rate variability. The shorter the sleep latency, the worse the alertness immediately after the 90-min nap. The 120-min and 30-min naps also revealed that prolonged total sleep time led to increased fatigue and drowsiness upon awakening. From 04:00 to 09:00, in the No-nap and One-nap groups, fatigue was higher than in the Two-nap group. The One-nap and Two-nap groups did not show improved morning performance. These results suggest that a split nap might improve drowsiness and fatigue during a long night shift.

## Introduction

Naps might be a beneficial and more natural way of sleeping for shift workers^1,2^. Naps are often taken during night shifts, and are reportedly associated with improved memory and learning ability, increased alertness, and improved mood^3^. Furthermore, naps lead to the maintenance of arousal levels^4,5^, which might enhance safety by reducing the risk of human error, and can be expected to have beneficial health effects by preventing disruption of the circadian rhythm if the nap is taken close to the circadian low of body temperature. The healthcare industry in Japan, and nursing in particular, represents a typical example of an industry that has increasingly adopted a shift work system. Since 2017, the number of nurses working 16-h night shifts in Japan has increased significantly^6^, which has resulted in increased physical and mental burden. Nurses working on such schedules often experience morning drowsiness and fatigue during their shift^7,8^. In addition, it has been reported that long working hours are associated with a higher risk of errors and accidents and poorer performance^9^.

Night work has been reported to increase morning drowsiness because of disruptions to the circadian rhythm^10^. Several studies have indicated that napping during a night shift can reduce drowsiness and maintain the efficiency of workers^2,11–14^.

A brief nap of 30 min or less has been demonstrated to lead to improved objective and subjective alertness, in addition to improvements in fatigue and vigilance^15^. Sixty-min naps, on the other hand, are associated with the problem of residual drowsiness due to sleep inertia. To minimize sleep inertia, a single sleep period of 90 min (i.e., one cycle) is considered appropriate^16^. Therefore, additional sleep before the effects of a nap of 90 min or longer wear off might help maintain the level of wakefulness^17^.

Informally, however, nursing staff at public hospitals are usually allowed to sleep or rest for up to 2 h during 16-h night shifts^18^, and many nurses avail of this nap during the night shift^19^. However, it is difficult to sustain the beneficial effect of a single monophasic nap for the remaining wakeful part of the shift. Split nap schedules might work better than monophasic sleep schedules for some individuals with a non-traditional work schedule, such as shift workers or on-call workers. In the case of a 16-h night shift, most nurses take a nap between 22:00 and 06:00^17^. Therefore, it is necessary to set naps in that time period during a 16-h night shift. Previous nap studies have examined sleepiness and sustained attention and performance by splitting sleep into night and daytime periods. However, no study has compared taking two naps during night shift hours to monolithic naps with continued data collection until completion of the night shift.

The author of this work previously co-researched the following nap conditions: a 120-min nap (start times: 22:00, 00:00, 02:00^20^) and a 90 + 30-min nap (start times: 22:30 + 02:30 and 00:30 + 04:30^21^). The results showed that the effects of naps are influenced by the time the nap is taken, and that the later the timing of taking a nap, the more effective it is in relieving morning drowsiness and fatigue. However, the previous research also clarified that when the nap is taken very late, it is associated with increased drowsiness before the nap. Hence, the ideal time for taking a nap and the ideal nap schedule during long night shifts need further elucidation. At this time, there is no scientific evidence that adopting a split nap schedule that limits total sleep is effective in maintaining optimal mental and physical health compared to a monophasic nap.

The aim of this study, which used data from previous studies^17,18^, was to investigate alertness and cognitive performance. immediately after and until the end of the night shift after taking a 120-min monophasic nap (One-nap) or a split 90-min and 30-min nap (Two-nap) during a 16-h simulated night shift, and the relationship between sleep quality and each of alertness and performance. It was hypothesized that under the conditions of biological night and extended wakefulness, a split nap would lead to a prolonged sleep effect, reduce drowsiness and fatigue, and improve performance when compared to a monophasic nap.

## Results

### Participants

Considering that many nurses are women, the participants in this study were 41 healthy adult females (mean age ± SD, 21.7 ± 0.9 years (n = 15) in the One-nap group, 22.2 ± 0.4 years (n = 14) in the Two-nap group, and 21.7 ± 0.5 years (n = 12) in the No-nap group). All participants were non-obese (body mass index [BMI] ≤ 25), and were recruited between August and November 2012, 2014, and 2018. There were no significant differences in age, BMI, or questionnaire scores between participants in the three groups, as shown in Table 1.

**Table 1:** Participants characteristics in the three groups.

	One-nap	Two-nap	No-nap	df(2, 38)	p
Age (years)	21.7 (0.9)	22.2 (0.4)	21.7 (0.5)	2.046	0.143
BMI	21.7 (2.2)	20.9 (2.2)	19.8 (2.4)	2.532	0.093
Question	51.5 (5.4)	54.7 (8.5)	49.9 (6.8)	1.637	0.208
Waking time (h:min)	09:04 (61)	08:14 (60)	07:57 (64)	4.504	**0.018**

Mean (standard deviation). Comparison of the waking times of the participants in the three groups showed that the One-nap group woke up much later than the No-nap group (*p* = 0.019). On the other hand, wake up time in the Two-nap group was not significantly different from those in the One-nap and No-nap groups.

*BMI* body mass index, *Question* morningness–eveningness questionnaire, *Waking time* average time the participants awoke on the morning of the study.

Significant differences between nap groups are highlighted in bold.

### Waking time for participants on the day of the experiment

The average time at which the participants awoke on the morning of the study were: 09:04 (SD: 61 min) in the One-nap group, 08:14 (SD: 60 min) in the Two-nap group, and 7:57 (SD: 64 min) the No-nap group. Comparison of the waking times of the participants in the three groups showed that the One-nap group woke up much later than the No-nap group (*p* = 0.019). On the other hand, waking time in the Two-nap group was not significantly different from those in the One-nap and No-nap groups.

### Sleep state during the experiment

Total sleep time (TST) was significantly longer with the 120-min nap as compared to the 90-min nap and the 30-min nap considered separately (93.1 min versus 68.4 and 20.1 min, respectively; *p* < 0.001), although there was no significant difference between the 120-min nap and the combined 90 + 30 min naps (93.1 min versus 88.5 min; *p* = 0.666). The sleep efficiency (SE; %) was not significantly different between the 120-min, 90-min, and 30-min naps [95.0 (8.7), 96.2 (8.7), and 99.1 (1.9), respectively; *p* = 0.556). Likewise, for sleep onset latency (SOL; min), there was no significant difference between the 120-min, 90-min, and 30-min naps [8.6 (13.6), 9.3 (12.5), and 5.8 (1.5), respectively; *p* = 0.721].

### Comparison of the three groups before and after naps

**Figure 1:**
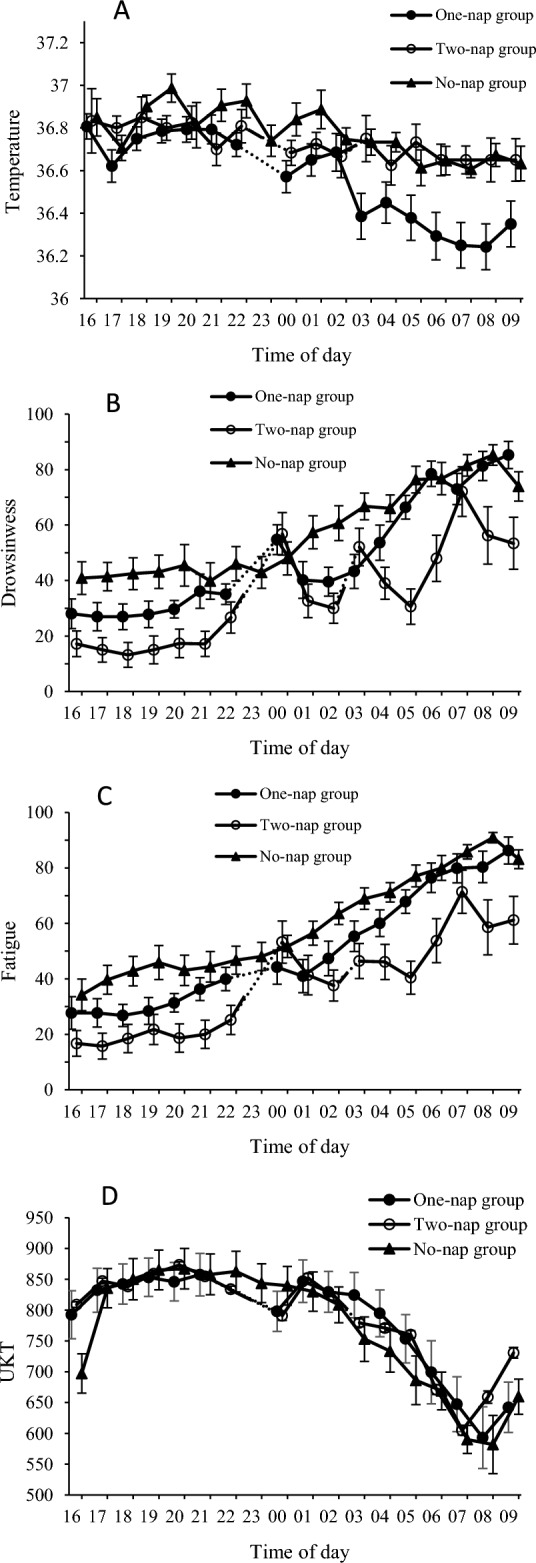
Temperature, drowsiness, fatigue and Uchida-Kraepelin test scores during asimulated night shift. The results represent the mean (± standard error) for bodytemperature (°C), subjective scales, and performance tasks. (**A**) Body temperature, (**B**) drowsiness, (**C**) Fatigue, (**D**) Uchida–Kraepelin test (UKT). Drowsiness and fatigue areshown as 0 values at 16:00.

**Figure 2:**
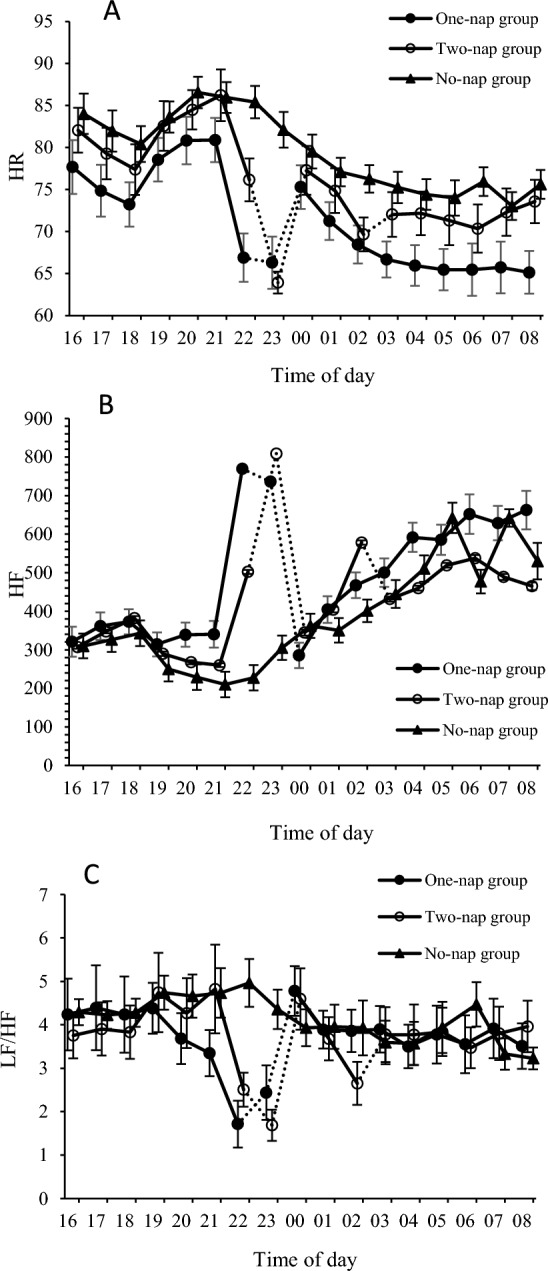
Heart rate (HR), high-frequency (HF) power of HRV, and low-frequency/high-frequency (LF/HF) ratio during a simulated night shift. Mean (± standard error) for HR, HF power and LF/HF ratio. (**A**) HR, (**B**) HF power, (**C**) LF/HF ratio.

Outcomes regarding the participants’ body temperature, visual analog scale (VAS), performance in the Uchida-Kraepelin test (UKT), and autonomic nervous activity from 16:00 to 09:00 are shown in Figs. 1 and 2. Table 2 shows the results of the analysis of neurobehavioral measures and autonomic activity between 21:00 or 22:00 and early morning, between 04:00 to 09:00.

**Table 2:** Linear mixed model analyses: physiological parameters, cognitive performance, and autonomic activity outcome measures between 21:00 or 22:00 and 04:00 to 09:00.

Outcome measure	Condition			Time			Condition*time		
	F df (2, 38)	*p*	*R2*	F df (6, 228)	*p*	*R2*	F df (12, 228)	*P*	*R2*
Temperature	7.178	**0.002**	0.274	12.484	** < 0.001**	0.247	1.604	0.092	0.078
Drowsiness	10.808	** < 0.001**	0.362	27.447	** < 0.001**	0.419	2.723	**0.002**	0.125
Fatigue	10.248	** < 0.001**	0.350	44.395	** < 0.001**	0.538	1.30	0.219	0.064
UKT (total score)	0.233	0.793	0.012	38.017	** < 0.001**	0.501	1.636	0.083	0.078

Outcome measure	Condition			Time			Condition*time		
	F df (2, 36)	*p*	*R2*	F df (5, 180)	*p*	*R2*	F df (10, 180)	*p*	*R2*
HR	3.529	**0.040**	0.163	153.595	** < 0.001**	0.810	2.721	**0.004**	0.131
LF/HF	0.121	0.886	0.006	2.559	**0.029**	0.066	2.550	**0.007**	0.124
HF	0.346	0.710	0.018	9.274	** < 0.001**	0.204	0.662	0.758	0.035

Results of linear mixed model analyses with main effects of condition: No-nap condition, One-nap condition, and Two-nap condition; between 21:00 or 22:00 and 04:00 to 09:00 (at 1-h intervals) and their interactions (condition*time).

*UKT* Uchida–Kraepelin test, *HR* heart rate, *LF/HF* low-frequency/high-frequency power ratio, *HF* high-frequency power, *F-static* variation between sample means, *df* degrees of freedom.

The text in bold indicates a significant difference (*p* < 0.05).

For body temperature, there were no significant differences between the three groups from 16:00 to 22:00. On the other hand, for post-nap, there was a significant main effect of condition (*p* = 0.002) and time (*p* < 0.001). Body temperature was significantly lower in the One-nap group than in the No-nap and Two-nap groups, and at the 04:00 point as compared to the 22:00 pre-nap point in all three groups (*p* < 0.001).

For pre-nap drowsiness, there was significantly less drowsiness reported by participants in the Two-nap group compared to the No-nap group (*p* = 0.001). There were no significant differences in drowsiness scores between the One-nap group and the other two groups. For post-nap, there was a significant main effect of condition (*p* < 0.001), time (*p* < 0.001), and a significant condition*time interaction (*p* = 0.002). In both the No-nap and One-nap groups, there was a significant increase in drowsiness from 04:00 to 09:00 (*p* < 0.05). For the Two-nap group, there was a significant increase in drowsiness at only 07:00 and 08:00 (*p* < 0.05).

In terms of pre-nap fatigue, the No-nap group reported significantly worse fatigue than the Two-nap group (*p* = 0.002), but there was no difference when compared to the One-nap group. On the other hand, there was a significant main effect of condition (*p* < 0.001) and time (*p* < 0.001) for post-nap, but with no significant condition*time interaction (*p* = 0.219). Participants in the Two-nap group experienced less fatigue than the No-nap group (*p* < 0.001) and the One-nap group (*p* = 0.002) from 04:00 to 09:00, while there was a significant increase in fatigue at 04:00 to 09:00 compared to 22:00 pre-nap in all three nap groups *(p* < 0.001).

As for pre-nap UKT (number of answers), there were no significant differences among the three groups. For post-nap, there was a significant main effect of time (*p* < 0.001) on performance in all three groups, such that all post-nap time scores were significantly worse than the 21:00 pre-nap scores.

In the One-nap group, electrocardiogram (ECG) data could not be obtained from one participant during the experiment; therefore, data were analyzed for a total of 40 participants: 14 participants in the One-nap group, 12 participants in the Two-nap group, and 14 participants in the No-nap group. There were no significant differences in pre-nap autonomic activity in heart rate (HR), high frequency (HF), and low frequency (LF)/HF ratio.

For post-nap heart rate data, there were significant main effects of condition (*p* = 0.040), time (*p* < 0.001), as well as condition*time (*p* = 0.004). Under all three conditions, heart rate decreased significantly during the period from 04:00 to 09:00 (*p* < 0.001).

For the LF/HF ratio, there were significant main effects of time (*p* = 0.029) and a significant condition*time interaction (*p* = 0.007). Under the One-nap group, there was no significant difference from 04:00 to 09:00 vs 21:00, and LF/HF ratio in the Two-nap group was significantly lower at 06:00 (*p* = 0.036) than at 21:00 (pre-nap). LF/HF ratio was significantly lower at 07:00–09:00 (*p* < 0.05) in the No-nap group.

There was a significant main effect of time (*p* < 0.001) on HF power. In all the groups, HF power at post-nap points (04:00 to 09:00) were significantly higher than those at 21:00.

### Sleep and subjective drowsiness, fatigue, and performance

Table 3 displays the results of correlational analyses of body temperature, subjective drowsiness, fatigue, and UKT with TST. Significant correlations were found for fatigue following the 120-min nap in the One-nap condition (Spearman’s correlation, r = 0.560; *p* = 0.037), and for drowsiness after the 30-min nap in the Two-nap condition (r = 0.589; *p* = 0.044). Briefly, with longer TSTs with the naps, there was a greater tendency for 120-min nap participants to report worse fatigue immediately after the nap, and for increased drowsiness to be reported immediately after the 30-min nap in the Two-nap group.

**Table 3:** Correlations between sleep parameters and changes in neurobehavioral outcomes from pre-nap to immediately after the nap.

Variable	One-nap	Two-nap	
	120-min nap	90-min nap	30-min nap
Total sleep time			
Temperature	− 0.146	0.529	− 0.051
Drowsiness	0.482	0.250	**0.589**
Fatigue	**0.560**	0.370	0.378
UKT	− 0.271	0.259	− 0.346
Sleep efficiency			
Temperature	− 0.182	0.556	− 0.057
Drowsiness	0.142	0.301	0.448
Fatigue	0.287	0.402	0.448
UKT	− 0.116	− 0.157	− 0.510
Sleep latency			
Temperature	0.064	− **0.589**	0.094
Drowsiness	0.042	− **0.614**	− 0.182
Fatigue	− 0.102	− **0.589**	− 0.140
UKT	0.433	0.549	− 0.010

Significant correlations are shown in bold.

*UKT* Uchida–Kraepelin test.

Correlational analyses did not show any significant correlation between sleep efficiency and body temperature, subjective drowsiness, fatigue, and UKT in each condition.

Sleep latency with the 90-min nap negatively correlated with body temperature, subjective drowsiness, fatigue, and UKT. Significant relationships were found between sleep latency and body temperature (r = − 0.589; *p* = 0.044), drowsiness (r = − 0.614; *p* = 0.034) and fatigue (r = − 0.589; *p* = 0.044) after the 90-min nap in the Two-nap condition. In other words, the shorter the sleep latency, the greater the tendency for a higher body temperature, increased drowsiness, and worse fatigue immediately after the 90-min nap.

## Discussion

This is the first study to investigate the effects of monophasic nap (One-nap: 120-min nap starting at 22:00) versus split naps (Two-nap: 90-min and 30-min naps starting at 22:30 and 02:30) taken at available time periods during a simulated 16-h night shift (16:00–09:00). The results suggested that towards the morning, the One-nap condition was associated with worse drowsiness from 04:00 to 09:00, and subjective fatigue was also increased compared to the Two**-**nap condition. On the other hand, the Two-nap condition reduced subjective drowsiness until 06:00 and fatigue until 09:00. This study found that a split nap ending at 03:00 helps to ameliorate the effects of extended drowsiness and fatigue. Furthermore, correlations investigating the association between TST, SE, SL, and subjective fatigue, drowsiness, temperature, and UKT, showed that sleep duration and sleep latency were associated with worse fatigue, drowsiness, and increased body temperature immediately after the 120-, 90-, and 30-min naps.

The degree of drowsiness is affected substantially if the body temperature reaches its lowest point during sleep or if the subject takes a long time to fall asleep^22^. In this study, the nap durations were set at 120 min, or 90 and 30 min for the One- and Two-nap conditions, respectively, and the net sleep state (TST, SE, and SOL) was the same. The split sleep group was predicted to have lower sleep pressure and poorer sleep quality, but the second nap was not different from the first nap in terms of SE or SOL. This suggests that short naps taken after prolonged awakenings may not reduce sleep pressure.

In addition, body temperature was significantly lower from 04:00 to 09:00 in the One-nap condition than in the No-nap condition and Two-nap conditions. Since there is a correlation between decreased body temperature and increased drowsiness^23,24^, the greater sleepiness with the monophasic versus the split nap was likely related to the lower body temperature following the monophasic nap. A 60- or 120-min nap taken between 23:00 and 02:00 has been found to reduce drowsiness through 04:00^25^. Hence, it appears that following the second 30-min nap, the effect of the nap might be expected to last for another 3 h^26^, excluding the temporary decrease in subjective alertness after the nap^16^.

In the Two-nap condition on the other hand, subjective drowsiness was maintained at the level of 22:00 until 06:00, and subjective fatigue was maintained at the 22:00 level until 09:00. HF power, which indicates the parasympathetic component of autonomic nerve activity, increased from 04:00 to 09:00 in all participants, both with and without a nap. In other words, this indicates that the parasympathetic nerve activity becomes dominant toward early morning, along with a subjective increase in drowsiness and fatigue, irrespective of whether or not the subject takes a nap. On the other hand, compared with the value at 21:00 of the LF/HF ratio, which reflects sympathetic nerve activity, the value decreased after 07:00 in the No-nap condition, with no change in the One-nap condition, and a decrease at 06:00 in the Two-nap condition. Usually, the LF/HF ratio increases sharply from the early morning hours before waking, to 1–2 h after waking^27^. In the case of a 120-min monophasic nap, the level at 21:00 was maintained in the early morning, indicating a change corresponding to the circadian rhythm. The evidence suggests that sleep windows of less than 30 min do not have a significant effect on performance^28^. Moreover, if increases in drowsiness are to be expected from 07:00 to 08:00, it might be necessary to add a 30-min nap between 05:00 and 06:00.

In the present study, correlations investigating the association between TST, SE, SL, and neurobehavioral outcomes revealed that the 120-min nap was associated with worse fatigue immediately after the nap with the longer TST, and for the 30-min nap, longer TST worsened drowsiness immediately after nap. On the other hand, increased body temperature, and worse drowsiness and fatigue were seen immediately after the nap following the 90-min nap, along with short SL. Naps of 90–120 min usually contain all sleep stages, including rapid eye movement (REM) and deep slow wave sleep (SWS), which helps to improve memory recall and recoup lost sleep. Cousins et al.^29^ set sleep opportunities at 8 or 6.5 h each day, with episodes at night or split between night sleep and a 90-min afternoon nap. They found that after taking a 90-min afternoon nap, factual knowledge learning in the afternoon benefited from split sleep, irrespective of sleep duration. The results of the UKT in the present study showed no effect of a 90-min nap plus a 30-min nap on early morning computational performance. The UKT is used to measure cognitive task performance, and involves mental arithmetic and handwriting^30^. It is possible that a 90-min nap may be more effective than a 30-min nap for complex task performance. Moreover, a previous study^31^ reported that a 200-min nap taken later in the night (04:00–08:00) significantly reduced memory performance compared to 200-min nap taken earlier in the night (00:00–04:00). As such, these results highlight and support that fact that later starts to naps should be avoided. A nap that is long enough to include a full sleep cycle, of at least 90 min, limits sleep inertia by allowing awakening from REM sleep. Sleep inertia refers to the grogginess and disorientation felt upon first waking from a nap^32^. Sleep inertia lowers the alertness level, which can lead to mistakes and even serious accidents. Some studies suggest that sleep inertia can be exacerbated by the amount of SWS in the prior sleep period, or by waking from SWS^33^. Therefore, the appropriate duration of a nap during the night shift when considering the effect of sleep inertia differs depending on whether the person wakes up before or after reaching SWS. However, although this study was not designed to evaluate sleep waves and effects of SWS, naps taken at a relatively early time at night, naps of 90 to 120 min, either at shorter SL or longer TST, were associated with increased subjective drowsiness and fatigue at awakening. This suggested that the participants may have awakened during stage 2 non-REM sleep. On the other hand, a second nap of 30 min increased sleepiness on awakening as sleep duration approached 30 min, possibly due to a delay in the onset of general SWS by 30 min^33–35^, since a longer sleep time would result in reaching SWS. However, it should be noted that the small sample size of this study limits the interpretation of these findings. A previous study showed that easier tests are more sensitive to sleep inertia than more demanding tests. Therefore, the use of a simpler test was also necessary to confirm sleep inertia^31^. Based on the results of the present study, during a night shift (e.g., from 16:00 to 09:00), a split nap (90 min and 30 min: ending at 00:00 and 03:00) is thought to be more effective than a monophasic nap (120 min: ending at 00:00) taken during work hours when tasks requiring quick responses to maintain a high level of safety are scheduled between 02:00 and 09:00.

During this time period, circadian alertness is low^36^ and the risk of accidents is increased. Therefore, a greater effect on the level of safety can be expected with the 90 min and 30 min split nap protocol compared with the 120 min monophasic nap. The importance of effective measures to reduce the drowsiness and fatigue experienced by shift workers is highlighted by the decreased alertness in the early morning shown in the No-nap condition and the One-nap condition throughout this study. Disruptions to the natural sleep–wake cycle can negatively impact health. While it might be more beneficial to get a single block of sleep of adequate length, it might also be beneficial to add a nap, if possible, if one cannot get a full block of sleep.

### Limitations

This study has several limitations. First, it was conducted under laboratory conditions; therefore, the timing of meals^37^ and the degree of change in performance and drowsiness might vary from those under actual working conditions, depending greatly on differences in workload due to the nature of the work or the timing of busy work periods. To confirm effective measures for reducing drowsiness and fatigue tailored to the conditions of specific professions and workplaces, interventional studies need to be conducted in actual workplaces. It is possible that the present results would be different if actual shift workers had been studied; therefore, caution is needed when interpreting the results. Second, in this study, although all the participants actually did sleep for the time they were asked to, based on the study protocol, some of them took a longer time to fall asleep. Finally, the participants in this study were all young women in their 20 s with no shift work experience, which might also have affected the results. I would like to investigate this issue further in a future study.

## Conclusions

This study reanalyzed existing pilot studies to determine the effects of 120-min monophasic nap or 90- and 30-min split naps during a simulated 16-h night shift. The results showed that taking a split nap during the night shift reduced drowsiness until 06:00 in the morning as well as for pre-nap at 22:00. Additionally, a split nap resulted in less fatigue than the monophasic nap until 09:00. However, the split and monophasic naps did not result in improved morning performance. The 120-min and 30-min naps also revealed that prolonged TST led to the appearance of increased fatigue and drowsiness upon awakening, while a shorter sleep latency increased fatigue and drowsiness after the 90-min nap. However, the levels of fatigue and drowsiness seen in this study may need to be interpreted with caution, as they may be different in an actual work environment. The results of this study are significant in that they can be used to inform how we address sleepiness and fatigue during limited rest periods in actual 16-h-long night shifts.

## Materials and methods

### Study design and participants

This study used and re-analyzed data from studies conducted from 2012 to 2018; the One-nap condition experiment was performed in 2012, the Two-nap condition experiment was performed in 2014, and the No-nap condition experiment was performed in 2018^37^.

None of the participants had any previous night shift experience, and none were identified as being morning type or evening type individuals according to the morningness–eveningness questionnaire^38^. All the participants were current nonsmokers, and since previous studies reported that subjective sleep quality deteriorates in the late luteal phase^39,40^, it was confirmed that none of the participants were in the luteal phase of their menstrual cycle at the time of the study, and had normal sleep patterns (habitual sleep ranging between 7 and 9 h). None of the participants were concurrently taking any prescription medications. The required total sample size was determined to be 36 participants (actual power 82.2%) based on an effect size, α error, and power (1 − β) of 0.25, 0.05, and 0.8, respectively. The power calculation in this study was carried out using G*Power 3^41^.

### Study protocol

**Figure 3:**
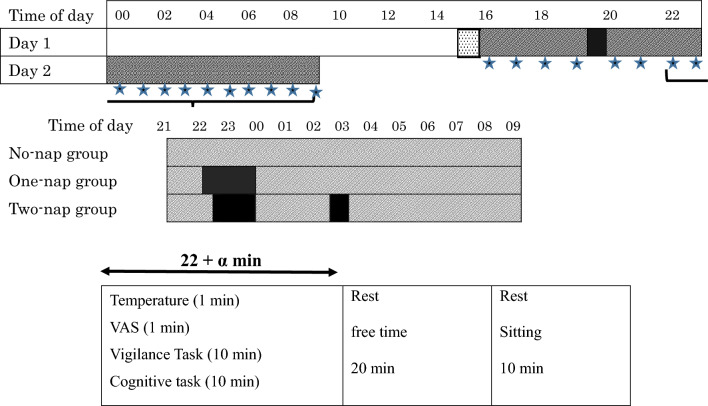
Schematic of the study protocol and experimental schedule. Each row in the upper panel represents 24 h. The gray hatched area shows the three nap conditions during the study period. The dotted area in the upper panel between 15:00 and 16:00 indicates the time of practice measurements, and the darker gray area represents meal times. The black areas in the lower panel show the nap schedules in the three groups. Protocol for temperature, subjective tests (VAS), Vigilance task (PVT), and cognitive task (UKT). All tests were measured every one hour (★). Simulated night-shift task. The night-shift task consisted of a 22-min task involving subjective and behavioural measures and a 30-min rest. *VAS* visual analogue scale (sleepiness and fatigue).

The protocol and experimental schedule of this study are shown in Fig. 3. The measurements were conducted over two consecutive days, between 16:00 on the first day and 09:00 the next day. From the time of commencement of the experiment, all participants wore an actigraphy monitoring device (ActiGraph; Ambulatory Monitoring Inc., Ardsley, NY, USA) on their non-dominant wrist and recorded their sleep and activity levels in a diary.

On the day of the experiment, all participants arrived at the laboratory at 15:00. They performed practice assessments until 16:00, including the UKT. At the start of each experiment, the participants were fitted with a heart rate variability (HRV) sensor to measure the R-R interval during the experiment using a 3-lead ECG system (GMS Inc., Tokyo, Japan). Every hour throughout the experiment, they were given 20 min to record their sublingual temperature once and complete the VAS for drowsiness and fatigue, and 10 min to perform the UKT. The next 20 min was considered free time, and the remaining 10 min was considered a rest period. The participants spent their free time reading, drawing, or drinking water. During the 10-min rest period, they sat on chairs and chatted with the other participants. The experiment started at 16:00 and ended at 09:00. The HRV sensor was removed at the end of each experiment, but the participants were asked to continue wearing the actigraphy monitoring device until they woke up on the day after the experiment. During the entire waking time of the experimental protocol, the participants remained awake in the laboratory and were continuously monitored by the researchers.

The three experimental conditions evaluated were no nap (No-nap), a monophasic nap from 22:00 to 00:00 (One-nap), and a split nap from 22:30 to 00:00 and 02:30 to 03:00 (Two-nap). All the participants stayed in a windowless and sound-insulated laboratory continuously over the 16-h experimental period. The laboratory environment was maintained at 26 ± 2 °C^42^ and 50% relative humidity. The participants sat on a chair with a backrest and worked at a desk, and the light intensity was maintained at 200 lx above the desk during the entire wakeful period of the experiment. When taking a nap, the participants went to a separate bedroom adjacent to the laboratory and lay down on the bed. They could adjust the level of ambient brightness/darkness during all scheduled sleep periods according to their preference.

Four to five participants were evaluated on each experimental day. All participants were instructed to refrain from strenuous physical exercise and consumption of caffeine or alcohol for 24 h prior to and during each study day. A researcher observed the participants and performed measurements from the start of the experiment to its completion. At the end of each scheduled nap time, the researcher notified the participants that it was time to wake up.

### Measurements

#### Sleep parameters

The sleep parameters measured during the 16 h simulated shift and the day after the shift included TST, SE (TST / time in bed × 100), and SOL. All parameters were measured using the actigraphy monitoring device.

#### Body temperature

The circadian rhythm of body temperature is one of the most frequently used indicators of circadian rhythmicity^43^, and body temperature has been shown to be related to drowsiness, fatigue, and performance on a single-digit mental arithmetic task^44^. Sublingual temperature, which is considered an index of internal body temperature^45^, was measured hourly using an oral thermometer (MC-612; Omron Inc., Kyoto, Japan) to assess changes in circadian modulation during the night.

#### Subjective drowsiness and fatigue

A VAS was used for the subjective assessment of drowsiness and fatigue^46^. The participants rated their drowsiness and fatigue every hour on a 100-mm line, with values ranging from 0 mm (not sleepy or tired at all) to 100 mm (extremely sleepy and tired).

##### UKT

The UKT (Nisseiken, Tokyo, Japan), a serial mental arithmetic task, was used to measure cognitive performance. This test is a questionnaire that requires intense concentration and effort, and has been used as a tool to induce mental stress^47^. The test material consisted of a pre-printed paper with 20 rows of 115 random, single-digit figures. The participants’ task was to add adjacent figures horizontally, and then write the one-digit answer between the two figures; they were asked to proceed along each row as quickly and as accurately as they could in a 1-min period. Upon being given the first cue, the participants began calculating from the first row. Then, when the second cue was given after 1 min, the participants were required to begin a new row, irrespective of their position on the current row. This procedure was repeated eight more times, for a total duration of 10 min. Each correct answer was given a score of one point. The sum of the scores for each 1-min period over the 10-min task (the highest score for 10 min was 2,280) was used as the value for the analysis. The researcher checked the number of correct answers and scored them.

#### Autonomic nervous system activity

For the purpose of the present study, HRV obtained through autoregressive analysis of R–R intervals were measured between 16:00 and 09:00. All data were analyzed offline after analog-to-digital conversion of 250 Hz R–R waves. HRV was automatically measured every 5 min during each hour and the values were averaged; these measurements were used to monitor autonomic nervous activity throughout the night^48^. HF power and the LF/HF ratio are used as indicators of cardiac parasympathetic and cardiac sympathetic nervous activity, respectively^49,50^. The LF/HF power ratio indicates the balance between sympathetic and parasympathetic outflows^51^.

### Statistical analysis

All results are shown as the mean ± standard deviation (SD) or standard error of the mean. All sleep variables measured on the day before the experiment were analyzed using one-way analysis of variance (ANOVA).

To test the effects of taking naps on neurobehavioral and physiological outcomes during early morning measurement periods, a fully saturated, linear mixed-effects analysis of variance was performed^52^, with a between-participant fixed effect of condition and a within-participant fixed effect of condition (No-nap, One-nap, Two-nap) and time (at 21:00 or 22:00 vs. from 04:00 to 09:00). Within-condition comparisons were used to minimize the effect of individual differences. Multiple comparisons were assessed using the Bonferroni correction to evaluate patterns of change under the three conditions.

Spearman’s correlation was used to determine the potential relationships between the sleep state of napping, subjective measurements, and autonomic nervous system activity whenever applicable, and the significant results are reported here. *P*-values below 5% were considered to be statistically significant in this study. All statistical analyses were performed using SPSS (version 22.0J; IBM, Tokyo, Japan). Effect sizes [(partial) R^2^-values] were calculated using the r2glmm package.

### Ethical considerations

This study used and re-analyzed data from previous studies. The experimental protocols of each of the previous studies were approved by the ethics committee, which additionally approved the secondary use of the data in this study. This study was approved by the Center for Integrated Medical Research of Hiroshima University (approval number: E-2221; approval on October 5, 2020). Written, informed consent was obtained from all participants before study participation. The study protocol conformed to the principles laid out in the Declaration of Helsinki. The author confirms that all ongoing and related trials for this intervention have been registered (Supplementary Information S1).

## Supplementary Information

Supplementary Information.

## Data Availability

The datasets used or analyzed during the current study are available from the corresponding author on reasonable request.
